# Effectiveness of a Supportive Telephone Counseling Intervention in Type 2 Diabetes Patients: Randomized Controlled Study

**DOI:** 10.1371/journal.pone.0077954

**Published:** 2013-10-30

**Authors:** Ute Mons, Elke Raum, Heike U. Krämer, Gernot Rüter, Dietrich Rothenbacher, Thomas Rosemann, Joachim Szecsenyi, Hermann Brenner

**Affiliations:** 1 Division of Clinical Epidemiology and Aging Research, German Cancer Research Center (DKFZ), Heidelberg, Germany; 2 Practice of General Medicine, Benningen/Neckar, Germany; 3 Institute of Epidemiology and Medical Biometry, Ulm University, Ulm, Germany; 4 Department of General Practice and Health Services Research, University Hospital of Zürich, Zürich, Switzerland; 5 Department of General Practice and Health Services Research, University Hospital Heidelberg, Heidelberg, Germany; Iran University of Medical Sciences, Iran (Republic of Islamic)

## Abstract

**Objectives:**

This randomized controlled trial investigated whether a patient-centered supportive counseling intervention comprising monthly telephone-based counseling sessions by practice nurses over 12 months improved diabetes-related medical and psycho-social outcomes above usual care in type 2 diabetes patients with poor glycemic control at baseline (HbA_1c_ >7.5%) in a primary care setting.

**Research Design:**

Patients were individually randomized into intervention (n = 103) and usual care group (n = 101). The primary outcome was change in HbA_1c_-concentration after 12 and 18 months. Secondary outcomes were lipid levels, blood pressure, health-related quality of life and symptoms of depression. Follow-up-measurements were carried out after 6, 12 and 18 months to assess potential immediate and maintained effects of the intervention. For the multivariate analysis, hierarchical linear models were computed for each outcome to assess within-group changes in outcomes over time and between-group differences in patterns of change.

**Results:**

HbA_1c_ (in %) decreased significantly from baseline to 12-month follow-up measurement both in the intervention (−0.44) and the usual care group (−0.51), but there was no significant between-group intervention effect. Significant improvements in the intervention group along with significant between-group differences were seen for health-related quality of life and, transiently, for systolic blood pressure and depression.

**Conclusions:**

Although we found no beneficial effect of the supportive telephone counseling in terms of a reduction of HbA_1c_ above usual care, our findings suggest some beneficial effects on cardiovascular risk factors, quality of life and depression. Continuous efforts might be needed to sustain improvements in patient outcomes.

**Trial Registration:**

ClinicalTrials.gov NCT00742547

## Introduction

Type 2 diabetes mellitus is a highly prevalent chronic disease especially in developed countries, and predictions indicate that prevalence will continue to increase worldwide [1]. In Germany, the overall prevalence of diabetes mellitus is around 7 to 9% [2], [3], and between 80 and 90% of these cases are type 2 [3]. Type 2 diabetes mellitus is associated with a high burden of related co-morbidities and complications, curtailing quality of life and increasing the risk of premature mortality [4], [5], and thus leading to considerable economic and health care costs [6]. The public health and economic burden associated with type 2 diabetes mellitus and related complications underlines the need for high quality diabetes care to sustainably improve patient health outcomes.

In order to achieve good glycemic control and to avoid complications, key components of outpatient diabetes care comprise patient-centered chronic disease management and support of patient self-management [7]–[9]. General practitioners (GPs) are central in chronic diabetes care but have only limited time per patient. These circumstances call for time- and cost-effective as well as easy-to-implement routines in general care. It has been suggested that patient care could be intensified without increasing time load for the GP by enhancing involvement of practice nurses [10], [11]. With regards to supporting lifestyle modifications and risk factor management, which is also crucial in type 2 diabetes care, telephone counseling has been shown to be effective in other specific patient groups [12], [13]. Therefore, supplemental supportive telephone-based counseling by practice nurses could be a feasible and cost-effective method to enhance the quality of medical care and to improve patients’ risk factor profile and associated outcomes.

The specific objective of this randomized-controlled trial (RCT) was to investigate whether a patient-centered intervention comprising monthly supportive telephone-based counseling sessions by practice nurses in a general practice setting improves diabetes-related medical and psycho-social outcomes above usual care in type 2 diabetes mellitus patients with poor glycemic control at baseline, who are at increased risk for many diabetes-associated complications [14].

## Research Design and Methods

### Ethics Statement

The study protocol for this trial was approved by the Ethics Committees of the Medical Faculty of the University of Heidelberg and by the State Chamber of Physicians of Baden-Württemberg. The protocol and supporting CONSORT checklist are available as supporting information; see Protocol S1 (English), Protocol S2 (German) and Checklist S1.

### Study Design, Participants and Randomization

The RCT is registered at clinicaltrials.gov (NCT00742547). The RCT was conducted in a subsample of participants of the DIANA study (“DIANA – Type 2 diabetes mellitus: New approaches to optimize medical care in general practice”), which is a prospective cohort study with patients with type 2 diabetes in general practices located in the area of Ludwigsburg/Heilbronn (South-West-Germany) [15]. In brief, adult patients with diagnosed type 2 diabetes mellitus were recruited by 38 participating general practices during regular practice visits from October 2008 to March 2010. Exclusion criteria for patients were living in a nursing home, insufficient knowledge of the German language, and visiting the GP for palliative or emergency care only. Participation was conditional on written informed consent. Overall, 1,146 type 2 diabetes patients participated at baseline.

Originally, it was planned to collaborate with 10 recruiting practices, as set out in the study protocol. However, after the start of the recruitment period it became evident that both the number of collaborating practices as well as the recruitment period had to be extended substantially in order to achieve a sufficient quantity of participants.

**Figure 1:**
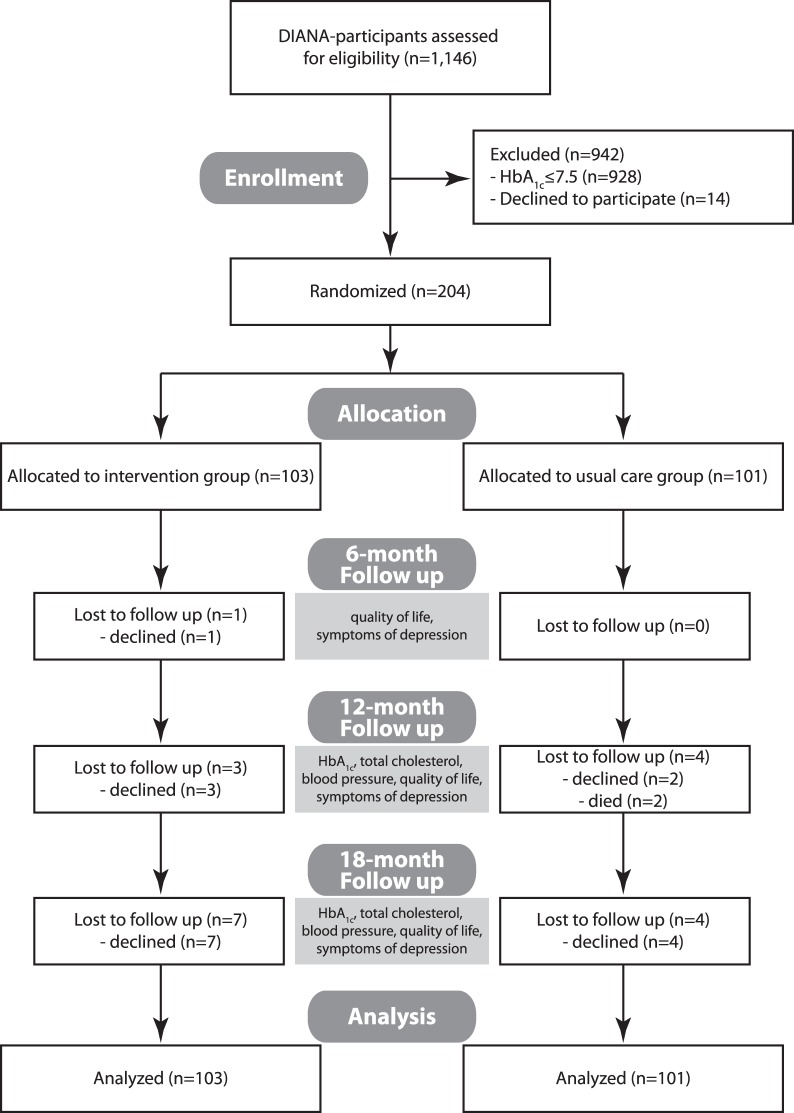
Flow diagram of the progress through the trial phases.

At the DIANA-study baseline examination, blood samples were taken from all participants by the GPs and HbA_1c_-concentration was determined by a contracted central laboratory. Since the intervention focused on patients with poor glycemic control, patients with a baseline glycosylated hemoglobin A_1c_ (HbA_1c_) >7.5% (equating 58.47 mmol/l) were eligible for the trial. Overall, 218 were eligible, of whom 204 (94%) gave written informed consent to participate in the trial and were randomized to intervention or control group (Figure 1). As it was originally planned to collaborate with only 10 GP practices, an individual randomization approach was chosen. A web-based randomization service for clinical trials provided by the Institute for Medical Informatics, Statistics and Documentation at the Medical University of Graz/Austria (http://www.randomizer.at) was used for the randomization. The minimization method was employed to balance group allocation by the predefined patient factors GP practice, sex and age (age <65 vs. age ≥65). 103 patients were randomized into the intervention group and 101 into the control group. After randomization, 3 patients (2.9%) of the intervention group declined to receive the intervention. 4 other patients (3.9%) never received the intervention for other reasons. 96 patients (93.2%) of those assigned to the intervention group actually received the intervention.

To evaluate immediate and long-term effects of the intervention, follow-up assessments of medical outcomes were carried out after 12 months (end-of-intervention) and 18 months (6 months post-intervention), and psycho-social outcomes were additionally measured after 6 months (during intervention). Of the 204 participants of the trial, 200 (99.5%) completed the first follow-up, 196 (96%) completed the second follow-up, and 185 (91%) also completed the third follow-up. The number of declines was somewhat higher in the intervention group (Figure 1).

### Intervention

The intervention was planned to consist of supportive telephone-based counseling sessions led by practice nurses (“Medizinische Fachangestellte”), who in Germany have obtained their professional qualification in a three-year dual vocational education.

After randomization, the GP practices were informed about the group allocation of their patients. Over the following 12 months, patients in the intervention group received supportive telephone-based counseling sessions on a monthly basis, which were conducted by practice nurses of the participating GP practices who had been trained beforehand. All of the sessions were conducted according to a written manual and were based on a standardized questionnaire developed by the authors. The development of the questionnaire relied on results from a qualitative study, for which interviews with GPs, practice nurses and patients had been conducted [16]. In brief, the final questionnaire included questions on the patient’s physical and mental condition, medication adherence, medical symptoms (cardiovascular symptoms, problems with the lower limbs, vision impairment), lifestyle (physical activity, weight, nutrition), and was designed to take about 10 minutes time overall. The items were designed to motivate patients to improve their general health behaviors, but also to identify barriers and problems regarding diabetes therapy and self-management on the part of the patients, and to facilitate early detection of diabetes-associated complications. In addition, the questionnaire sheet included guidelines on the identification of problems that required a resolution by the GP. In case of the patient’s answers being indicative of any physical or mental complications or issues that might compromise the success of diabetes therapy, the practice nurse was supposed to alert the GP, who then decided about further actions and contacted the patient himself, if necessary.

82 of the 89 patients (92%) who received telephone counseling and who did not drop-out during follow-up received 10 to 12 sessions. The other 7 (8%) patients received between 6 and 9 sessions.

Patients in the control group received usual care only, i.e. no study-related telephone-based counseling sessions or other systematic procedures.

### Measures and Outcomes

Primary outcome of this study was the change in HbA_1c_-concentration after 12 and 18 months. At baseline and at the 12- and 18-month follow-up measurements, blood samples were taken from the participants and sent to the contracted central laboratory which determined HbA_1c_-concentrations using ion exchange high pressure liquid chromatography (G8, Tosoh Biosciences). The inter-assay variation coefficients provided by the manufacturer are 0.7% at medium concentrations of HbA_1c_ (mean: 7.4%) and 0.36% at high concentrations (mean: 13.5%).

Secondary outcomes of interest were diastolic and systolic blood pressure, serum cholesterol level, health-related quality of life, and symptoms of depression as an indicator of mental health. Lipid levels (total cholesterol and HDL cholesterol) as well as blood pressure were obtained from the GPs, who provided up-to-date medical information about the patient from their medical records with a standardized questionnaire at baseline and at the 12- and 18-month follow-up measurements. Because of a comparably high number of missing values in HDL cholesterol levels (Table 1), only total cholesterol was used as an outcome in the multivariate analyses. GPs also reported patient’s age at first clinical diagnosis of type 2 diabetes mellitus.

**Table 1:** Baseline characteristics of study participants.

		Intervention group	Usual care group	*P*-value
*N*		103	101	
Age (in years)	median (IQR)	68.0 (17)	67.0 (15)	*P* = 0.60^a^
Sex	% Male	60.2	62.4	*P* = 0.75^b^
	% Female	39.8	37.6	
Duration of diabetes (in years)	median (IQR)	9.0±7.5	9.0±10.0	*P* = 0.97^a^
	*N* missing	7	7	
Marital status	% married	64.1	59.4	*P* = 0.49^b^
	% not married	35.9	40.6	
Education	% low	72.6	64.4	*P* = 0.11^b^
	% moderate	21.6	20.8	
	% high	5.9	14.9	
	*N* missing	1	–	
Diagnosis of coronary heart disease	% yes	24.8	24.0	*P* = 0.90^b^
	% no	75.3	76.0	
	*N* missing	2	5	
Diagnosis of diabetic nephropathy	% yes	19.2	11.7	*P* = 0.15^b^
	% no	80.8	88.3	
	*N* missing	4	7	
HbA_1c_ (in %)	median (IQR)	8.0 (0.9)	8.2 (1.1)	*P* = 0.15^a^
Total cholesterol (in mg/dl)	mean ± SD	194.6±41.7	193.4±44.7	*P* = 0.85^c^
	*N* missing	2	6	
HDL cholesterol (in mg/dl)	mean ± SD	45.8±12.2	51.0±24.3	*P* = 0.09^c^
	*N* missing	22	28	
Blood pressure (in mmHg)	Diastolic, median (IQR)	80 (5)	80 (14.5)	*P* = 0.44^a^
	Systolic, median (IQR)	140 (20)	135 (15.5)	*P* = 0.02^a^
	*N* missing	2	5	
Health-related quality of life (according toSF-12)	Physical component summary score,median (IQR)	42.6 (22.5)	46.5 (21.3)	*P* = 0.66^a^
	Mental component summary score,median (IQR)	53.3 (16.4)	53.8 (16.5)	*P* = 0.95^a^
	*N* missing	4	3	
Symptoms of depression	GDS sum score, median (IQR)	2 (4)	2 (5)	*P* = 0.79^a^
	*N* missing	3	3	

*Note: N* = number of cases, SD = standard deviation, IQR = interquartile range.

a According to Mann-Whitney U test.

b According to Chi-squared test.

c According to t-test.

Health-related quality of life (HRQoL) was assessed with the Short Form General Health Survey (SF-12) [17]. The item responses of the SF-12 were weighted and combined as described elsewhere [18] to obtain a physical and a mental component summary score, with higher scores representing greater physical and emotional well-being, respectively. Symptoms of depression were measured at baseline and all three follow-up measurements using the Geriatric Depression Scale (GDS) in the 15-item version [19]. A sum score was calculated giving the total number of self-reported symptoms of depression ranging from 0 to 15, with scores greater than 5 suggesting depression. Both the SF-12 and the GDS were part of self-administered questionnaires that participants filled in at baseline, and at 6-, 12- and 18-month follow-up. The baseline questionnaire also collected information on the socio-demographic background, such as year of birth, educational level and marital status. Highest level of education was translated into three categories (low, moderate, high) and marital status was dichotomized (being married versus not married).

### Statistical Analyses

The study was designed to achieve at least 90% power to detect differences in HbA_1c_-concentrations of 0.5%-points across groups at a significance level of 0.05 (two tailed); the minimum required sample size would be 68 patients per group. The final group size was substantially larger, as 218 patients of the cohort were eligible for the study and 204 of these gave informed consent to participate.

For by-group comparisons of categorical variables, Chi-square tests were employed. Continuous variables were tested for normal distribution using the Shapiro-Wilk-test. The t-test for independent samples was used for by-group comparisons of normally distributed continuous variables, and the Mann-Whitney U-test in case of non-normally distributed continuous variables.

For the multivariate analysis, hierarchical linear models (HLMs) were computed, which can handle multiple measurements, intra-individual correlation in observations over time, and the nested structure of the data (with GP practices as clusters) [20], [21]. For the analyses, each available measurement of each participant was treated as one single observation, i.e. there were up to four observations per participant. Separate models were computed for each outcome (all continuous), adjusted for sex, age, marital status and educational level (all fixed effects, time-invariant), and accounting for intra-individual correlation over time (first-order autoregressive covariance structure), and clustering by GP practice (random effect, time-invariant). To allow for non-linear development of outcomes, time was treated as a categorical variable with each measurement representing one category, and was included as time-varying fixed effect. Allocated group was included as time-invariant binary fixed effect. To test for between-group differences in within-group changes over time, a time-by-group interaction term was added as time-varying fixed effect. Estimates for within-group changes over time (with respective *P*-values) and *P*-values of between-group differences in within-group changes over time were reported for each outcome.

For the primary outcome HbA_1c_, sex-specific least square means based on the parameters estimated in sex-stratified multivariate HLMs were also graphically depicted.

All analyses were based on the initial treatment intent (intention to treat analyses). All statistical tests were two-sided, with an alpha level of 0.05. SAS v9.2 was used throughout.

## Results

Median age in the intervention and control group was 68 and 67 years and slightly more than 60% of participants were male in both groups (Table 1). Participants in intervention and usual care group did not differ significantly with regards to socio-demographic characteristics. Regarding medical characteristics, systolic blood pressure was significantly higher in the intervention group.

HbA_1c_ decreased significantly from baseline to 12-month follow-up measurement (Table 2) both in intervention and usual care group (by 0.44 in the intervention and by 0.51 in the usual care group). However, patterns of change did not differ significantly between groups. In contrast to the usual care group, the reduction in HbA_1c_ was not maintained in the intervention group with values measured at baseline and at 18-month follow-up not being significantly different. Again, patterns of change in this period did not differ significantly between groups.

**Table 2:** Within group changes in medical outcomes over time and between-group differences.

	Baseline to 12-month follow-up			Baseline to 18-month follow-up		
	Within-group changes in outcome over time	*P*-value of between-group changes (time by group)		Within-group changes in outcome over time	*P*-value of between-group changes (time by group)	
***HbA_1c_ (in %)***						
**Intervention group**	−0.44 (*P*<0.001)	*P* = 0.70		−0.22 (*P* = 0.12)		*P* = 0.19
**Usual care group**	−0.51 (*P*<0.001)			−0.49 (*P*<0.001)		
***Total cholesterol (in mg/dl)***						
**Intervention group**	0.54 (*P* = 0.88)		*P* = 0.26	0.23 (*P* = 0.96)	*P* = 0.43	
**Usual care group**	−5.27 (*P* = 0.17)			−5.09 (*P* = 0.30)		
***Diastolic blood pressure (in mmHg)***						
**Intervention group**	−1.80 (*P* = 0.13)		*P* = 0.66	0.03 (*P* = 0.98)	*P* = 0.94	
**Usual care group**	−1.05 (*P* = 0.68)			−0.11 (*P* = 0.75)		
***Systolic blood pressure (in mmHg)***						
**Intervention group**	−5.27 (*P* = 0.007)		*P* = 0.007	−1.76 (*P* = 0.44)	*P* = 0.27	
**Usual care group**	2.35 (*P* = 0.25)			1.84 (*P* = 0.44)		

*Note.* Results of separate hierarchical linear models for each outcome. Models were adjusted for sex, age, marital status and education, were specified to take into account intra-individual correlation over time and clustering by GP practice (random effect), and included time and time×group-interaction effects. Within-group changes in outcomes over time and respective *P*-values, and the *P*-values of the time×group (between-group) effect are reported.

For total cholesterol and diastolic blood pressure, neither change over time in either group nor between-group differences in patterns of change were statistically significant. Systolic blood pressure significantly decreased from baseline to 12-month follow-up in the intervention group only (by −5.27), with between-group differences in patterns of change being significant. The decrease was however not maintained with systolic blood pressure at 18-month follow-up not being significantly different from baseline and with patterns of change not being statistically significant between groups.

For the physical HRQoL (Table 3), values did not change significantly in either group from baseline to 6-month or to 12-month follow-up, and patterns of change were also not statistically different between groups. However, patterns of change from baseline to 18-month follow-up varied between groups due to a significant increase by 3.02 in the intervention group, while physical HRQoL decreased insignificantly in the usual care group. The mental HRQoL decreased significantly in the usual care group by 3.21 points from baseline to 18-month follow-up. At the same time, mental HRQoL tended to remain stable in the intervention group. However, group differences in patterns of change were not statistically significant over this period.

**Table 3:** Within group changes in psycho-social outcomes over time and between-group differences.

	Baseline to 6-month follow-up				Baseline to 12-month follow-up		Baseline to 18-month follow-up			
	Within-group changes in outcome over time	*P*-value of between-group changes(time by group)			Within-group changes inoutcome over time	*P*-value of between-group changes	Within-group changes in outcome over time		*P*-value of between-group changes (time by group)	
***Health-related quality of life, SF-12 physical component summary score***										
**Intervention group**	0.91 (*P* = 0.28)		*P* = 0.61	0.92 (*P* = 0.40)		*P* = 0.86		3.02 (*P* = 0.017)	*P* = 0.018	
**Usual care group**	0.29 (*P* = 0.74)			0.66 (*P* = 0.55)				−1.23 (*P* = 0.33)		
***Health-related quality of life, SF-12 mental component summary score***										
**Intervention group**	1.04 (*P* = 0.23)		*P* = 0.06	−0.32 (*P* = 0.77)		*P* = 0.59		−0.34 (*P* = 0.79)	*P* = 0.11	
**Usual care group**	−1.24 (*P* = 0.16)			−1.17 (*P* = 0.29)				−3.21 (*P* = 0.01)		
***Symptoms of depression (GDS sum score)***										
**Intervention group**	−0.41 (*P* = 0.04)		*P* = 0.007	−0.13 (*P* = 0.64)		*P* = 0.65		0.06 (*P* = 0.86)	*P* = 0.47	
**Usual care group**	0.35 (*P* = 0.08)			0.05 (*P* = 0.87)				0.39 (*P* = 0.24)		

*Note.* Results of separate hierarchical linear models for each outcome. Models were adjusted for sex, age, marital status and education, were specified to take into account intra-individual correlation over time and clustering by GP practice (random effect), and included time and time×group-interaction effects. Within-group changes in outcomes over time and respective *P*-values, and the *P*-values of the time×group (between-group) effect are reported.

Symptoms of depression changed significantly only during the first six months of the intervention, with patterns of change being significantly different between groups. The depression score decreased significantly by 0.41 in the intervention group, while it increased borderline significantly by 0.35 in the usual care group. The improvement in the intervention group was however not maintained.

**Figure 2:**
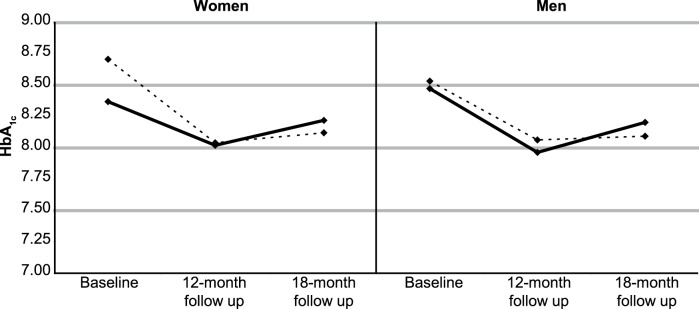
Least square means for HbA_1c_ at the different measurements by group and sex. *Legend:* solid line = Intervention group, dashed line = usual care group *Note.* Least square means are based on parameter estimates of sex-stratified hierarchical linear models for HbA_1c_. Models were adjusted for sex, age, marital status and education, were specified to take into account intra-individual correlation over time and clustering by GP practice (random effect), and included time and time×group-interaction effects.

Sex-stratification of models yielded overall consistent results for both sexes (details not shown for secondary outcomes; see Figure 2 for primary outcome HbA_1c_).

## Discussion

Our results from a randomized controlled trial of regular supportive telephone-based counseling sessions by practice nurses for diabetes patients with dissatisfactory glycemic control in a general practice setting suggest only limited effects of the intervention with respect to HbA_1c_-levels, the primary outcome measure, whereas positive effects were seen with some of the secondary outcomes. HbA_1c_-levels decreased significantly in the intervention group from baseline to end-of-intervention, but not above usual care. In addition, the decrease in the intervention group was partly reversed post-intervention. However, favorable changes were observed in several of the secondary endpoints. Systolic blood pressure decreased significantly in the intervention group and above usual care; however, the reduction was not maintained post-intervention. With regard to HRQoL as measured with the SF-12, we observed significant longer-term changes post-intervention for the physical component score, which improved significantly in the intervention group compared to baseline and above usual care. For symptoms of depression, beneficial effects of the intervention were observed in the first six months of the intervention but which were not maintained.

In general, primary care interventions involving revisions of professional roles such as enhanced involvement of nurses in diabetes management have been reported to have positive effects on patient outcomes [10], [22]–[25]. To our knowledge, only few intervention studies in primary care have used telephone-based counseling by practice nurses to improve outcomes in type 2 diabetes-patients in a GP setting, of which most proved to be effective in improving at least some patient outcomes, even though no general pattern seems to be apparent. A trial of nurse-led telephone counseling (cognitive behavioral therapy plus a walking program) over one year with about 170 patients per group has not led to improvements in HbA_1c_, but patients in the intervention group had greater improvements in systolic blood pressure, physical activity and depressive symptoms compared to controls [26]. A trial in 275 nearly exclusively male veterans with non-insulin-dependent diabetes designed to improve glycemic control with an intervention involving monthly nurse-led telephone calls over one year to educate patients, monitor their health status and facilitate access to primary care, could not find an improvement in coronary risk factors above the control condition [27]. However, as reported elsewhere, the same intervention led to modest improvements in glycemic control but not in health-related quality of life or in diabetes-related symptoms [28]. In a lifestyle intervention study in patients with hypertension, non-insulin-dependent diabetes or coronary heart disease, 212 patients either received usual care, or monthly telephone or face-to-face individual counseling by nurses for one year following one face-to-face individual counseling session. While significant improvements in dietary patterns were observed in all groups, the intervention did not result in reductions in fat intake, serum cholesterol or body mass index above usual care [29].

Overall, our findings are consistent with the literature suggesting some benefit of an enhanced involvement of practice nurses in diabetes care [10], [22]–[25]. Our findings are also in concordance with the aforementioned intervention studies that – albeit finding improvements in lifestyle-related and psycho-social outcomes – could not establish an added benefit to usual care in terms of distinct improvements of medical outcomes. We focused on medical and psycho-social outcomes in this study, but further analyses indicated no statistically significant beneficial intervention effect with regards to lifestyle risk factors such as smoking behavior or physical activity patterns (details not shown). Future follow-up measurements will show whether our supportive counseling intervention nonetheless exerted effects on the long-term prognosis, such as the development of secondary disorders, adverse events and mortality.

In the spirit of comparative effectiveness research, this study meant to be a pragmatic trial [30], [31]. It was conducted under real-life-conditions in the general practice routine with practice nurses employed at the cooperating practices conducting the counseling sessions, instead of having study counselors perform the intervention. Furthermore, it focused only on the challenging high-risk patient group of type 2 diabetes mellitus patients with poor glycemic control. To increase generalizability of the findings, apart from this selection as few exclusion criteria as possible were applied.

Although few patients allocated to the intervention group never received the intervention due to non-availability of patients or non-cooperation of single practice nurses, the progression of the intervention was overall satisfactory and proved to be applicable. In a process evaluation sheet, a large majority of the cooperating practice nurses rated the counseling as useful and reported being mostly or very satisfied with the time and effort that was involved with the telephone counseling. The vast majority of the patients on the other hand were also quite satisfied with the intervention (details not shown).

Some limitations need to be considered when interpreting the findings of this study.

Firstly, the patients were individually and not cluster-randomized to intervention and control group. This may have led to contamination bias, with the practice nurses conducting the telephone-based counseling or the GPs unknowingly enhancing care for patients in the usual care group. This might explain the short-term improvements that were observed in patients of the usual care group and would have led to an underestimation of the intervention effect. However, the reduction of HbA_1c_ that was observed in both groups could as well reflect a statistical artifact due to “regression toward the mean” [32]. This phenomenon describes the tendency of extreme values measured at one point to progress toward the population average in the following and has especially relevant explanatory power in this study which included only patients with increased baseline HbA_1c_-values.

Secondly, another issue is sample attrition. The comparably high initial participation rate (93.6%) and retention rate (90.7%) however suggest only limited potential for selection or attrition bias. The risk of attrition bias was further reduced by using all available data over the follow-ups in the linear models, i.e. participants lost to sample attrition were included until the last recorded measurement. Adding a binary variable to the models indicating drop-out at any follow-up measurement in sensitivity analyses did not change results (details not shown), supporting that drop-out did not distort our findings.

Thirdly, seven patients of the intervention group never received the intervention and were nevertheless included in all analyses examining the treatment effect (based on intention to treat). Excluding those cases from the models in sensitivity analyses resulted in only negligibly different parameter estimates and p-values (details not shown).

Time is a scarce resource in a busy GP practice environment and any measures to enhance the quality of care in a GP practice setting should be easy-to-implement and involve as little additional financial and time costs as possible. Therefore, the intervention was designed to be telephone-based and to use a standardized questionnaire instrument. Objectives of this instrument were monitoring of patients’ health status, identification of patient-side barriers to compliance with the treatment, and support of patients’ self-management of diabetes therapy. The questionnaire instrument and accompanying guidelines thus covered crucial elements of type 2 diabetes treatment and were designed to continuously inform and support the patients’ diabetes therapy and to complement usual care. An intervention individually tailored to patient’s needs might have proven to be more effective, but would have probably required more of the practice nurses’ time and effort.

In conclusion, this randomized controlled study could not show a beneficial effect of telephone counseling in terms of an improvement of HbA_1c_ in diabetes patients with increased HbA_1c_ at baseline. However, our findings suggest some beneficial effects on important diabetes-associated risk factors such as blood pressure, and on quality of life and mental health. That most of the improvements in outcomes were not maintained after the end of the intervention suggests that longer-term and continuous efforts are needed to sustain improvements in patient outcomes.

## Supporting Information

Checklist S1 **CONSORT Checklist.**
(DOCX)

Protocol S1 **Trial Protocol.**
(PDF)

Protocol S2 **Trial Protocol (German).**
(PDF)
