# Supplementary material for: Effectiveness of a Supportive Telephone Counseling Intervention in Type 2 Diabetes Patients: Randomized Controlled Study
Source: PLoS One. 2013 Oct 30;8(10):e77954. doi: 10.1371/journal.pone.0077954 (PMC3813502; doi:10.1371/journal.pone.0077954)
Supplement: Protocol S1 — Trial Protocol. (PDF) [file pone.0077954.s002.pdf]

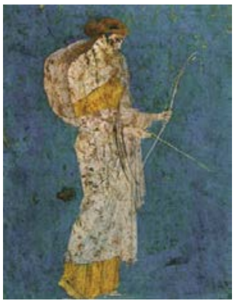

DIANA

Diabetes mellitus: Neue Wege der Optimierung der allgemeinärztlichen Betreuung

(Diabetes mellitus: New Approaches to Optimize Medical Care in General Practice)

Study protocol

(Version: 1. October 2008, translated into English: 15. May 2013)

Project leader, responsible study doctor:

Prof. Dr. med. Hermann Brenner, Dr. med. Elke Raum

German Cancer Research Center (DKFZ)

Division Clinical Epidemiology and Aging Research

Bergheimer Str. 20

69115 Heidelberg

Germany

Summary

Patients with type 2 diabetes mellitus have a high risk for a multitude of severe complications. This epidemiological cohort study aims at examining determinants and predictors of long-term prognosis in about 1,500 patients with type 2 diabetes mellitus, who are recruited and followed up in a general practitioner setting. A randomised controlled trial will be conducted in a subgroup of around 250 patients with poor glycemic control ($HbA_{1c} > 7.5\%$), in order to investigate whether supportive patient-centred telephone counselling improves the primary outcome HbA_{1c} and secondary outcomes (complications, hospital admissions, mortality) above usual care.

Index of contents

Summary 2

1. Introduction 4

2. Objectives of the study/Outcomes 4

3. Procedure to be examined 5

3.1 General description 5

Study part A: Epidemiological cohort study 5

Study part B: Randomised controlled trial 5

3.2 Effects (therapeutic, diagnostic) 5

3.3 Unintended effects, other risks, strains for participants 5

4. Study design 6

Study part A 6

Study part B 6

5. Randomisation procedure 6

Study part A 6

Study part B 6

6. Inclusion and exclusion criteria 7

7. Course of the study 7

Study part A 7

Study part B 9

Analysis of blood parameters 9

8. Accompanying therapy 10

9. Security laboratory (if relevant) 10

10. Termination criteria 10

10.1 Individual termination criteria 10

10.2 Termination criteria for the study 11

11. Statistical design 11

12. Ethical and legal aspects 14

Signatures 15

References 16

Appendix 17

1. Introduction

Diabetes mellitus is one of the most prevalent metabolic diseases in Western industrial countries. General practitioners (GPs) are usually the main care takers of diabetes patients (1). The goals of diabetes therapy are based on evidence-based guidelines (2). In Germany, currently around 5 million people (6 % of the general population) have type 2 diabetes and prevalence is expected to increase in the future (3, 4).

The quality of diabetes care determines prognosis in diabetes patients. Especially the willingness of affected patients to engage in an adequate patient self-management is of great importance. Although current epidemiological studies have shown some improvements in diabetes care and therapy at a population level, still a considerable proportion of patients have a poor glycemic control (5). Furthermore, population-based epidemiological longitudinal data on determinants and predictors of long-term prognosis of diabetes patients in Germany are very sparse.

Patient-centred diabetes care that incorporates patients' resources can improve important outcome parameters of diabetes therapy. Successful diabetes management, amongst others, is based on a close collaboration of GP and patient that considers personal coping strategies of the patient, and on a good patient self-management (6, 7). However, only little is known about which factors and structures determine successful patient self-management.

2. Objectives of the study/Outcomes

This epidemiological cohort study aims at examining determinants and predictors of long-term prognosis in patients with type 2 diabetes mellitus in a GP setting. A randomised controlled trial will be conducted in a subgroup of patients with poor glycemic control, in order to investigate whether supportive patient-centred telephone counselling improves the primary outcome HbA_{1c} and secondary outcomes (diabetes-related complications, diabetes-related hospital admissions, mortality) above usual care. The study will also examine how eligible patients unwilling to participate in the intervention study differ from participants with regards to basic characteristics and outcomes and whether this has an impact on the generalisability (external validity) of the study's findings.

3. Procedure to be examined

3.1 General description

Study part A: Epidemiological cohort study

Epidemiological cohort study to examine determinants and predictors of long-term prognosis in patients with type 2 diabetes mellitus (1,500 patients) in GP care over a period of 20 years.

Study part B: Randomised controlled trial

As procedure to be examined, telephone counselling will be conducted as described in the following:

I. Intervention group:

- The intervention group will receive telephone counselling, which will be carried out by trained nurses of the participating GP practices.
- By using a patient-oriented questionnaire patients will be called in regular intervals (every 4 to 6 weeks, approx. 10 times) over a period of 12 months, and will be questioned and counselled regarding diabetes-related aspects.
- Each counselling session will take approx. 15 minutes.
- The usual diabetes therapy (as taken care of by the GP) will be continued independent of the telephone counselling.

II. Control groups (patients randomised to control group, and eligible patients unwilling to participate in the intervention study):

- Patients in the control group will receive usual care, but will not receive telephone counselling sessions.

3.2 Effects (therapeutic, diagnostic)

Objective of the study is to clarify whether and to what extent the concept of care in patients with type 2 diabetes mellitus with poor glycemic control can be improved.

3.3 Unintended effects, other risks, strains for participants

A blood sample will be collected from all participants at the beginning of the study, after 12 months (only from participants of the intervention study) and after 18 months. To further

minimise the low risk associated with the venous puncture, the blood collection will be conducted in the context of a venous puncture in GP routine care, if possible.

Unnecessary strains for the participating patients will be prevented by providing assistance for specific problems and giving participants the opportunity to withdraw their consent to participate at any time.

4. Study design

Study part A

Epidemiological cohort study in patients with type 2 diabetes mellitus

Study part B

In a sub-group of cohort study participants additionally: Open randomised controlled trial with about 124 patients each in intervention and control group. According to the „comprehensive cohort design", patients who are eligible but do not agree to participate will be followed up as non-randomised control group (no telephone counselling; approx. 64 patients).

5. Randomisation procedure

Study part A

Not applicable – observation study.

Study part B

In order to prevent a distortion of results by potential confounders, patients will be individually randomised. The randomisation will be conducted centrally in the study centre at the DKFZ for all eligible patients of the participating GP practices. The web-based patient randomisation service for multi-centre clinical trials of the Institute for Medical Informatics, Statistics and Documentation at the Medical University of Graz, Austria, will be used for the randomisation (http://www.randomizer.at). Because of the limited number of study participants and the number of participating GP practices, the minimisation method is used to ensure an equal distribution of potential influencing factors in the two randomised study arms. As influencing factors the GP practice (as study centre) and age and sex of the participants will be considered.

6. Inclusion and exclusion criteria

Inclusion criteria:

- Type 2 diabetes mellitus patients who are in diabetes care at one of the 10 collaborating GP practices in the Ludwigsburg area during the period of recruitment (presumably from 01.10.2008 – 31.12.2008),
- Ability and willingness to participate in the study.

For study part B additionally:

- HbA_{1c} > 7,5% (as being determined centrally at entrance to the cohort study for all participants).

Exclusion criteria:

- Insufficient knowledge of the German language,
- Nursing home resident,
- Palliative care situation with limited life time,
- Patients seeing the GP for emergency care or by way of exception only.

7. Course of the study

Study part A

During the recruitment period, all patients with type 2 diabetes mellitus of the participating practices will be informed about the planned study. Of diabetes patients giving consent to participate in the study (estimated number: N=1500) a blood sample will be drawn to determine HbA_{1c} in order to check for eligibility for study part B (inclusion criterion: HbA_{1c} > 7.5%). HbA_{1c} will be determined by a central external laboratory in the Ludwigsburg-area for all patients in order to guarantee the comparability of this essential study parameter. At this point, patients will receive the patient questionnaire by their GP, which they are asked to fill in at home and send to the Division Clinical Epidemiology and Aging Research at DKFZ. The HbA_{1c}-results will be passed on promptly to the Division Clinical Epidemiology and Aging Research at DKFZ by the external laboratory. The parts of the blood samples that are not needed for the determination of HbA_{1c} will be collected and frozen in the laboratory and then collectively sent to the Division Clinical Epidemiology and Aging Research at DKFZ for long-term storage and later analyses.

In order to investigate determinants and predictors of long-term prognosis in patients with type 2 diabetes mellitus, the following information relevant for the project will be collected by patients and their attending GPs at baseline as well as at all follow-up measurements (over a period of up to 20 years; first follow-up: after 18 months):

Standardised patient questionnaires:

Covering the following topics (amongst others):

− quality of life: e.g. SF12, EQ5D,

− sense of coherence: SOC13,

− patient assessment of chronic illness care (PACIC 5A, German version),

− diabetes-related disorders,

− blood glucose control,

− life style (including smoking, alcohol consumption, nutrition, physical activity),

− depression: e.g. German version of the Patient Health Questionnaires (PHQ9).

At baseline measurement, the following topics will be additionally covered:

− previous diabetes-related patient education;

− socio-demographic variables: education, marital status, occupation.

Standardised GP questionnaires:

• Recruitment questionnaire:

This questionnaire will be filled in by the attending GP for all patients at inclusion into the study. Especially the following topics will be documented:

− detailed disease history,

− diabetes: duration of disease, previous therapy, complications,

− comorbidity,

− medication at recruitment,

− laboratory results at recruitment,

− type of diabetes care: disease management program or usual care,

− appraisal of patient’s compliance.

• Follow up-questionnaires:

The attending GP documents the following topics (among others) for all patients at all follow-up measurements:

− incident diseases (especially diabetes-complications),

− laboratory results (amongst others: fasting blood glucose, lipids, creatinine, albumin in urine),

− findings of physical examination (amongst others: body weight, blood pressure, pulse, sensory functions),

− medication,

− diabetes-related referrals to specialists (e.g. ophthalmologists, nephrologists),

− inpatient hospital admissions and their medical causes.

At the beginning of the study structural characteristics of the participating practices will be collected using a standardised questionnaire. These are in particular:

− type of practice: single or group practice, shared practice,

− year of establishment of the practice,

− additional qualifications,

− number of patients, share of private patients.

Study part B

Patients fulfilling the additional inclusion criterion for study part B will be either randomised into one of the two study arms of the trial or – if they do not consent to participate in the trial – will be assigned to the second control group. The intervention group will receive telephone counselling sessions every 4 to 6 weeks during the first 12 months after recruitment. The intervention and control groups will additionally receive standardised patient questionnaires (see above) after 6, 12 and 18 months. The diabetes therapy during the course of the intervention study, including occasioned diagnostic and therapeutic measures as well as incidence of potential diabetes-related complications (e.g. myocardial infarction) and hospital admissions, will be documented using the standardised follow-up GP questionnaires (see above) after the end of the intervention (at 12 and 18 months).

After completion of study part B the patients will be also followed-up in the context of the epidemiological cohort study.

Analysis of blood parameters

The measurement of the primary outcome (HbA_{1c}) and other parameters will be conducted in all participants (study part A and B) at recruitment and follow-ups.

In participants of study arm B an additional measurement of the primary outcome will be conducted after 12 months (end of intervention).

The blood collection will be carried out in the participating practices. Overall about 30 ml blood per patient will be drawn (EDTA + serum). The handling of the blood samples in the practices, the shipping to and the analyses conducted by the central laboratory in the Ludwigsburg-area as well as the storage of the blood samples will be carried out according to standard operating procedures.

Those blood samples that are not immediately needed for the determination of HbA_{1c} will be shipped and transferred to the Division Clinical Epidemiology and Aging Research at DKFZ for the scientific investigation of further markers (including genetic markers) that could be important for the onset and progress of diabetes mellitus and potential complications. The samples will be preserved without time limit, in order to allow for specific laboratory analyses with improved technical possibilities and considering new scientific findings in the future. The biological material that will be preserved without time limit will be completely anonymised after completion of data collection to render inference to the individual study participant impossible. If necessary, specimens will be shipped to external project-related collaborating partners with specific expertise in special laboratory analyses that cannot be conducted at the DKFZ. Commercial utilisation interests will be excluded for that matter.

8. Accompanying therapy

Not relevant.

9. Security laboratory (if relevant)

Not necessary.

10. Termination criteria

10.1 Individual termination criteria

- withdrawal of consent,
- inability to continue participation,
- In the case of withdrawal from the study the respective participant has the right to demand the complete elimination of his pseudonymised data. For this, all possibilities of

withdrawal will be listed and explained and separately asked at the time of withdrawal.

10.2 Termination criteria for the study

- lack of willingness to participate among practices,
- insufficient recruitment of patients,
- for the trial no interim analysis is intended that could justify a preliminary termination of the study.

11. Statistical design

Study part A:

The statistical examination particularly includes the following analyses:

- a thorough description of the whole cohort regarding parameters relevant for diabetes (amongst others: age, sex, duration of disease, medication, body weight, comorbidity, life style factors) at recruitment,
- epidemiological analyses regarding determinants and prognostic markers in patients in the overall cohort after completion of further follow-up measurements.

Study part B:

Power calculation:

Assuming that both in the intervention and in the control group about 4 patients are lost in the course of the study (n=120 per group), with 2-tailed testing, equal variance, a standard deviation of 1.0, a significance level of $\alpha=0.05$, the following statistical power results regarding the decrease in HbA_{1c} (see next page):

**Decrease of HbA_{1c} of ... %-points in the intervention group**	**Power**
0,1	0,12
0,2	0,34
0,3	0,64
0,4	**0,87**
0,5	**0,97**
0,6	**0,99**
0,7	**1,00**
0,8	**1,00**
0,9	**1,00**
1,0	**1,00**

The proposed study therefore has sufficient power to detect clinically relevant changes in HbA_{1c} with the selected sample size.

The scientific evaluation will investigate the following outcomes:

Primary outcome: ***HbA_{1c}***

HbA_{1c} is the main outcome of diabetes therapy and reflects the quality of metabolic control in the past three months.

HbA_{1c} will be determined at the beginning of the study as well as immediately and 6 months after the end of the intervention in all trial participants.

Secondary outcomes:

Diabetes mellitus is a chronic disease in which the further course of health (incidence of complications and sequelae) largely depends on a preferably integrated medical care as well as on lifestyle factors. Therefore the following secondary outcomes will be evaluated besides the primary outcome HbA_{1c}:

- lipid metabolism control
- blood pressure
- body weight, physical activity, smoking
- health-related quality of life
- incidence of diabetes-related complications

- hospital admissions
- mortality.

The primary working hypothesis is that supportive telephone counselling in these patients helps improving the primary outcome of diabetes therapy (HbA_{1c}). The secondary working hypothesis is that, as consequence, also secondary outcomes (diabetes-related complications, hospital admissions, mortality etc.) will improve.

The statistical examination includes the following analyses:

- a thorough description and comparison of control and intervention group regarding diabetes-related parameter (amongst others: age, sex, duration of diabetes, medication, body weight, comorbidity, life style factors),
- a thorough description and comparison of control and intervention group regarding the primary and secondary outcomes at the end of the intervention,
- analyses on the association of intervention and changes in outcomes during long-term follow-up (sustainability of the intervention),
- a thorough description and comparison of eligible patients unwilling to participate in the trial and of control and intervention group regarding the primary and secondary outcomes at the end of the intervention (assessment of external validity/generalisability of results).

The comparison of change in HbA_{1c} in both study groups using multiple linear regression and adjusting for potential confounders will be the main analysis of the intervention study (study part B).

Further analyses will be conducted, also adjusting for potential confounders and using multivariate regression models (multiple linear regression or Cox proportional hazards models, depending on the outcome). These will be based on a priori formulated protocols for analyses. All analyses will be conducted using the statistical software SAS and under the supervision of the project leader.

Gender specific aspects will be considered in sub-group analyses by doing sex-stratified analyses. That way, different factors can be considered in the multivariate models, regarding which men and women may differ (health behaviour, perceptions of illness/health, etc).

12. Ethical and legal aspects

The investigation will be conducted in accordance with the Declaration of Helsinki in the current version from 1996, as well as on the guidelines for Good Epidemiological Practice (GEP) adopted by the German Working Group for Epidemiology (Deutsche Arbeitsgemeinschaft für Epidemiologie, DAE) that are based on the Declaration of Helsinki, and according to the code of medical ethics of the Chamber of Physicians of the state Baden-Württemberg in its respective current versions. The participation of patients in the study is voluntary. The consent to participate can be withdrawn at any time, for any reason and without any disadvantages for further medical care. The study does not involve any risks for its participants. The participants have sufficient time to look into the objectives and process of the study at home. They have the chance to pose questions to the study secretariat or the participating GPs. After their questions have been answered, subjects willing to participate may sign the written informed consent form.

The nature and consequences and especially the possible benefit and potential risks of the planned study will be explained to the patients before beginning of the study both in written and oral form. Their consent will be documented with a signature on the informed consent form. At withdrawal from the study the patient is entitled to request the elimination of already collected (data) material.

Before the beginning of the study, the study protocol will be submitted to the ethics commission of the Medical Faculty of the University of Heidelberg for review. Inclusion of patients/subjects will not start before having received a written affirmative vote from the ethics commission.

The names of the patients and all other confidential information are subject to medical confidentiality and the regulations of the German data protection law (Bundesdatenschutzgesetz, BDSG). If necessary, a transfer of patient data will only occur in pseudonymised form. Commercial utilisation interests will be excluded for that matter.

Patient questionnaires, informed consent forms and transfer contract

The questionnaires to be filled in by study participants carry a unique ID number. Study participants will be asked to fill in name, address and date of birth on the cover page of the questionnaire. After receipt of the questionnaire at the DKFZ the cover page with the person-identifying information will be separated from the rest of the questionnaire, which carries the ID number only. The further storage and processing of person-identifying data will be performed completely separately from the processing of the study data.

The person-identifying information on the cover page will be processed on a stand-alone

personal computer in the study secretariat, which is not connected to a network. This computer provides programs and files for the management of the study participants. Only staff in charge of study organisation has access to this computer. The computer is secured with special security software (Safeguard Easy), for which a password is needed to gain access.

After removal of the personal ID number, the cover page of the questionnaire will be stored exclusively in a separate steel locker in a room with special locking system in the study secretariat. Only a limited and nominated number of persons will hold a key to this room.

The informed consent form and the transfer contract will include name and signature of the study participant. After receipt at DKFZ, they will be stored exactly as described for the cover page of the questionnaire (see above).

Blood samples

The blood samples will be collected, shipped, stored, processed and analysed exclusively in pseudonymised form (after the end of data collection only in anonymised form), i.e. only tagged with the ID number.

Signatures

Prof Dr. med. H. Brenner Dr. med. Elke Raum

References

(1) Rothenbacher D, Brenner H, Rüter G. Netreuung von chronisch Kranken in der Hausarztpraxis am Beispiel des Typ 2 Diabetes. Dtsch Ärztebl 2005;102:A 2408-2412.

(2) Nationale Versorgungs-Leitlinie Diabetes mellitus Typ-2. URL: http://www.versorgungsleitlinien.de/themen/diabetes2 (letzter Zugriff: 16.04.2008).

(3) Rathmann W, Haastert B, Icks A, Herder C, Kolb H, Holle R, et. Al. Die Diabetes-Epidemie in der älteren Bevölkerung in Westeuropa: Daten aus bevölkerungsbezogenen Studien. Gesundheitswesen 2005;67 (S1):S110-S114.

(4) Stock S, Redaelli M, Wendland G, Civello D, Lauterbach KW. Diabetes-prevalence and cost of illness in Germany: a study evaluating data from the satutory health insurance in Germany. Diabet Med 2006;23:299-305.

(5) Icks A, Rathmann W, Haastert B, Mielck A, Holle R, Löwel H, et. al. Versorgungsqualität und Ausmaß von Komplikationen an einer bevölkerungsbezogenen Stichprobe von Typ 2-Diabetespatienten. Dtsch Wochenschr 2006;131:73-78.

(6) International Diabetes Foundation. International standards for diabetes education 2003. http://www.idf.org/webdata/docs/International%20standards.pdf (letzter Zugriff: 16.04.2008).

(7) Campbell SM, Hann M, Hacker J, Burns C, Oliver D, Thapar A, et. al. Identifying predictors of high quality care in English general practice: observational study. BMJ 2001;323:1-6.

Appendix

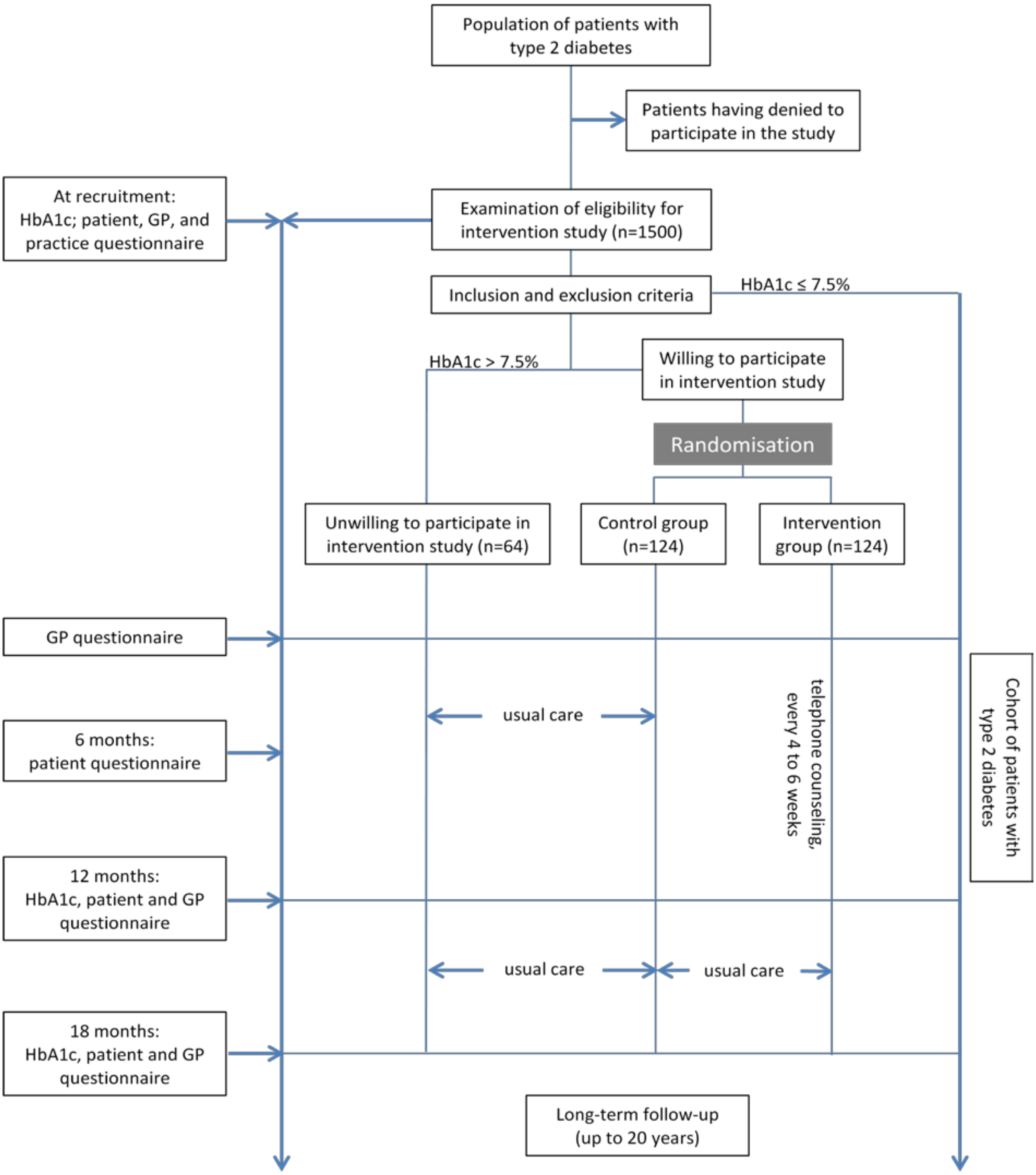

Population of patients with type 2 diabetes
Patients having denied to participate in the study
At recruitment: HbA1c; patient, GP, and practice questionnaire
Examination of eligibility for intervention study (n=1500)
Inclusion and exclusion criteria
HbA1c ≤ 7.5%
HbA1c > 7.5%
Willing to participate in intervention study
Randomisation
Unwilling to participate in intervention study (n=64)
Control group (n=124)
Intervention group (n=124)
GP questionnaire
usual care
telephone counseling, every 4 to 6 weeks
Cohort of patients with type 2 diabetes
6 months: patient questionnaire
12 months: HbA1c, patient and GP questionnaire
usual care
usual care
18 months: HbA1c, patient and GP questionnaire
Long-term follow-up (up to 20 years)
