# Supplementary material for: Effectiveness of a Supportive Telephone Counseling Intervention in Type 2 Diabetes Patients: Randomized Controlled Study
Source: PLoS One. 2013 Oct 30;8(10):e77954. doi: 10.1371/journal.pone.0077954 (PMC3813502; doi:10.1371/journal.pone.0077954)
Supplement: Protocol S2 — Trial Protocol (German). (PDF) [file pone.0077954.s003.pdf]

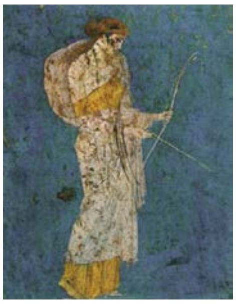

DIANA

Diabetes mellitus: Neue Wege der Optimierung der allgemeinärztlichen Betreuung

Studienplan

(Version vom 01.10.2008)

Leiter der Studie, verantwortlicher Studienarzt:

Prof. Dr. med. Hermann Brenner, Dr. med. Elke Raum

Deutsches Krebsforschungszentrum in der Helmholtz-Gemeinschaft

Abteilung Klinische Epidemiologie und Alternsforschung

Bergheimer Str. 20

69115 Heidelberg

Zusammenfassung

Patienten[1] mit Diabetes mellitus Typ 2 haben ein hohes Risiko für eine Vielzahl schwerwiegender Folgeerkrankungen. In einer epidemiologischen Längsschnittstudie sollen Determinanten und Prädiktoren der Langzeitprognose bei zirka 1500 Patienten mit Typ 2 Diabetes untersucht werden, die im Kontext der allgemeinärztlichen Betreuung rekrutiert und nachbeobachtet werden. In einem Subkollektiv von zirka 250 Patienten mit unbefriedigender Stoffwechsellage (HbA_{1c} > 7,5%) soll mittels einer randomisierten Interventionsstudie untersucht werden, ob ein supportives Telefoncounseling den Zielparameter der Diabetestherapie (HbA_{1c}) und andere Outcome-Parameter (Folgeerkrankungen, Krankenhauseinweisungen, Sterblichkeit) durch gezieltes patientenorientiertes Vorgehen im Vergleich zur „normalen" medizinischen Versorgung verbessert.

[1] Im Folgenden bezieht sich der Ausdruck Patient sowohl auf weibliche als auch männliche Patienten

Inhaltsverzeichnis:

Zusammenfassung **2**

1. Einleitung **4**

2. Ziele der Studie/Zielkriterien **4**

3. Zu prüfendes Verfahren **5**

3.1 Allgemeine Beschreibung *5*

Studienteil A: Epidemiologische Längsschnittstudie *5*

Studienteil B: Randomisierte Interventionsstudie *5*

3.2 Wirkungen (therapeutisch, diagnostisch) *5*

3.3 Unerwünschte Wirkungen, sonstige Risiken, Belastungen für den StudienteiInehmer *5*

4. Studiendesign **6**

Studienteil A: *6*

Studienteil B: *6*

5. Randomisierungsverfahren **6**

Studienteil A: *6*

Studienteil B: *6*

6. Ein- und Ausschlusskriterien **7**

7. Studienablauf **7**

Studienteil A: *7*

Studienteil B: *9*

Untersuchung von Blutparametern *9*

8. Begleittherapie **10**

9. Sicherheitslabor (soweit einschlägig) **10**

10. Abbruchkriterien **10**

10.1 Individuelle Abbruchkriterien *10*

10.2 Abbruchkriterien für die Gesamtstudie *10*

11. Statistisches Design **11**

12. Ethische und rechtliche Aspekte **13**

Unterschriften **15**

Literatur **15**

Appendix **16**

1. Einleitung

Der Diabetes mellitus gehört zu den häufigsten Stoffwechselerkrankungen in den entwickelten Industrieländern. Viele der betroffenen Patienten werden in erster Linie von hausärztlich tätigen Ärzten betreut (1). Die Therapieziele bei der Behandlung von Patienten mit Diabetes sind klar formuliert und basieren auf evidenzbasierten Leitlinien (2). In Deutschland sind derzeit ungefähr 5 Millionen Menschen (6% der Gesamtbevölkerung) betroffen und es ist davon auszugehen, dass die Häufigkeit in den nächsten Jahren deutlich steigen wird (3, 4). Die Prognose des Diabetes mellitus wird maßgeblich von der Versorgungsqualität bestimmt, die über die Jahre hinweg angeboten wird. Insbesondere die Bereitschaft der betroffenen Patienten zu einem krankheitsgerechten Verhalten ist von enormer Bedeutung. Aktuellen epidemiologischen Studien zufolge hat sich zwar die Versorgung, vor allem die Diabetes-Einstellung in den vergangenen Jahren bevölkerungsweit etwas verbessert, doch weist nach wie vor ein erheblicher Anteil der Patienten verbesserungswürdige Stoffwechselparameter auf (5). Bevölkerungsbezogene epidemiologische Längsschnittdaten zu Determinanten und Prädiktoren der Langzeitprognose von Diabetes-Patienten in Deutschland fehlen bisher weitestgehend.

Eine patientenorientierte, hausärztliche Betreuung chronisch kranker Patienten, die auch die Ressourcen des Patienten einbezieht, kann wesentliche Outcomeparameter einer Diabetes-Therapie verbessern. Erfolgreiches Diabetesmanagement basiert u. a. auf einer engen Kooperation von Arzt und Patient unter Berücksichtigung der persönlichen Coping- und Bewältigungsstrategien des Patienten sowie einem guten Selbstmanagement der Patienten (6, 7). Von welchen Faktoren und Prozessstrukturen ein gutes Selbstmanagement abhängt, ist bis dato noch zu wenig bekannt.

2. Ziele der Studie/Zielkriterien

In einer epidemiologischen Längsschnittstudie sollen Determinanten und Prädiktoren der Langzeitprognose von Patienten mit Typ 2 Diabetes im Kontext der häuslichen Betreuung untersucht werden. In einem Teilkollektiv von Patienten mit unbefriedigender Stoffwechsellage soll im Rahmen einer randomisierten Interventionsstudie untersucht werden, ob sich mithilfe eines supportiven Telefoncounselings sowie eines gezielten patientenorientierten Vorgehens die Zielparameter der Diabetestherapie (HbA_{1c}) und andere sekundäre Outcomeparameter (diabetesbedingte Krankenhauseinweisung, Sterblichkeit, Inzidenz diabetes-assoziierter Folgeerkrankungen) im Vergleich zur gegenwärtigen medizinischen Versorgung verbessern lassen. Zudem wird untersucht, wie sich die Patienten, die sich im Rahmen der Interventionsstudie randomisieren lassen, von denen, die sich nicht randomisieren lassen, hinsichtlich der Basischarakteristika und Outcomeparameter unterscheiden und welche Auswirkungen dies auf die Generalisierbarkeit (externe Validität) der Studienergebnisse hat.

3. Zu prüfendes Verfahren

3.1 Allgemeine Beschreibung

Studienteil A: Epidemiologische Längsschnittstudie

Epidemiologische Längsschnittstudie zur Untersuchung von Determinanten und Prädiktoren der Langzeitprognose von Patienten mit Typ 2 Diabetes (1500 Patienten) in der allgemeinärztlichen Betreuung über einen Zeitraum von 20 Jahren.

Studienteil B: Randomisierte Interventionsstudie

Als das zu prüfende Verfahren wird ein Telefoncounseling wie im Folgenden beschrieben, durchgeführt:

I. Interventionsgruppe:

- Die Interventionsgruppe erhält ein Telefoncounseling, das von speziell geschulten medizinischen Fachangestellten der teilnehmenden Praxen durchgeführt wird.
- Mit Hilfe einer patientenorientierten Monitoringliste werden die Patienten in regelmäßigen Abständen (alle 4-6 Wochen, zirka 10 Termine) 12 Monate lang hinsichtlich wichtiger für den Verlauf des Diabetes mellitus relevanter Aspekte befragt und beraten.
- Das Counseling dauert ca. 15 Minuten.
- Die vom betreuenden Hausarzt durchgeführte Diabetesbehandlung wird unabhängig davon fortgesetzt.

II. Kontrollgruppen (randomisiert und Randomisierungs unwillig):

- Die Kontrollgruppen werden von den Hausärzten weiterhin der üblichen Behandlung unterzogen, erhalten aber kein Telefoncounseling.

3.2 Wirkungen (therapeutisch, diagnostisch)

Mit Hilfe der Studie soll geklärt werden, inwieweit sich das Versorgungskonzept von Patienten mit Typ-2 Diabetes, die bisher eine schlechte Therapietreue aufweisen, verbessern lässt.

3.3 Unerwünschte Wirkungen, sonstige Risiken, Belastungen für den Studienteilnehmer

Bei allen Studienteilnehmern erfolgt eine Blutentnahme zu Beginn der Studie und nochmals nach 12 (nur Teilnehmer der Interventionsstudie) und 18 Monaten. Um das geringe Risiko durch die Venenpunktion noch weiter zu minimieren, wird die Blutentnahme so weit als möglich im Rahmen ei-

ner in der hausärztliche Betreuung notwendigen Venenpunktion durchgeführt.

Unnötigen Belastungen für die Patienten, die an der Studie teilnehmen, wird vorgebeugt, indem Hilfestellungen zu spezifischen Fragestellungen gegeben wird und jederzeit die Einwilligung zur Teilnahme an der Studie zurückgezogen werden kann.

4. Studiendesign

Studienteil A:

Epidemiologische Kohortenstudie bei Patienten mit Diabetes mellitus Typ 2

Studienteil B:

Bei einem Teilkollektiv der Teilnehmer der Längsschnittstudie zusätzlich: Offene, randomisierte Interventionsstudie mit jeweils 124 Patienten in der Interventions- bzw. Kontrollgruppe. Gemäß des „comprehensive cohort design“ werden Patienten, die zwar die Einschlusskriterien erfüllen aber nicht randomisierungswillig sind, als nichtrandomisierte Randgruppe (kein Telefoncounseling; ca. 64 Patienten) mitbeobachtet.

5. Randomisierungsverfahren

Studienteil A:

Nicht zutreffend – reine Beobachtungsstudie

Studienteil B:

Zur Prävention der Verzerrung der Ergebnisse durch potentielle Störgrößen wird eine individuelle Randomisierung der Patienten durchgeführt. Die Randomisierung erfolgt zentral im Studienzentrum am DKFZ für alle Studienteilnehmer der beteiligten Praxen, die die Einschlusskriterien erfüllen. Zur Durchführung der Randomisierung wird der webbasierte selbst bedienbare Randomisierungsdienst (http://www.randomizer.at) für multizentrische klinische Studien des Instituts für Medizinische Informatik, Statistik und Dokumentation der Medizinischen Universität Graz, Österreich, genutzt. Aufgrund der begrenzten Zahl an Studienteilnehmern und der Anzahl an teilnehmenden Praxen wird mittels der Minimisierungsmethode eine Gleichverteilung potentieller Einflussfaktoren in den beiden randomisierten Studienarmen gesichert. Als Einflussfaktoren werden bei Randomisierung und Analyse zum einen die teilnehmenden Praxen (im Sinne von Studienzentren), zum anderen Alter und Geschlecht der Studienteilnehmer berücksichtigt.

6. Ein- und Ausschlusskriterien

Einschlusskriterien:

- Diabetiker, die während des Rekrutierungszeitraums (voraussichtlich: 01.10.2008 – 31.12.2008) in einer von 10 kooperierenden Hausarztpraxen im Raum Ludwigsburg betreut werden,
- Fähigkeit und Bereitschaft zur Teilnahme an der Studie.

Für Studienteil B zusätzlich:

- HbA_{1c} > 7,5% (wird bei Studienbeginn zentral für alle Diabetiker bestimmt).

Ausschlusskriterien:

- unzureichende Deutschkenntnisse,
- Heimbewohner,
- palliative Versorgungssituation mit begrenzter Lebenszeit,
- Notfall- oder Vertretungspatienten.

7. Studienablauf

Studienteil A:

Alle Patienten mit Typ-2 Diabetes mellitus der teilnehmenden Praxen werden zur Beginn der Rekrutierungsphase von ihren Hausärzten über die geplante Studie informiert und aufgeklärt. Von den Diabetikern, die einwilligen (geschätzte Anzahl: N=1500), an der Studie teilzunehmen, wird eine aktuelle HbA_{1c}-Bestimmung durchgeführt, um das Einschlusskriterium für Studienteil B (HbA_{1c} > 7,5%) zu überprüfen. Die HbA_{1c}-Bestimmung wird einheitlich für alle Patienten und Praxen von einem zentralen Labor im Raum Ludwigsburg durchgeführt, um die Vergleichbarkeit dieses zentralen Studienparameters zu garantieren. Zu diesem Zeitpunkt erhalten die Patienten von ihrem Arzt den Patientenfragebogen, den sie zu Hause ausfüllen und an die Abteilung Klinische Epidemiologie und Alternsforschung senden. Die Blutparameter werden vom Labor an die Abteilung Klinische Epidemiologie und Alternsforschung zeitnah weitergeleitet. Die für die HbA_{1c}-Bestimmung nicht benötigten Teile der Blutproben werden zunächst im Labor gesammelt und eingefroren, um sie gebündelt an die die Abteilung Klinische Epidemiologie und Alternsforschung zur langfristigen Aufbewahrung für weitere Analysen zu senden.

Zur Untersuchung von Determinanten und Prädiktoren der Langzeitprognose von Patienten mit Diabetes mellitus Typ 2 werden folgende für das Projekt relevante Sachverhalte bei der Basiserhebung und bei allen Folgeerhebungen (über einen Zeitraum von bis zu 20 Jahren, 1. Folgeerhebung:

nach 18 Monaten) bei den teilnehmenden Patienten und behandelnden Ärzten erfragt:

Standardisierter Patientenfragebogen:

Er erfasst u.a. folgende Bereiche:

- Lebensqualität: z.B. SF12, EQ5D,
- Sense of Coherence: SOC13,
- Patient Assessment of Chronic Illness Care (PACIC 5A, deutsche Version),
- Diabetes-spezifische Beschwerden,
- Blutzuckerkontrolle,
- Lebensgewohnheiten (einschließlich Rauchen, Alkohol, Ernährung, körperliche Aktivität),
- Depressivität: z.B. deutsche Version des Patient Health Questionnaires (PHQ9).

Bei der Basiserhebung werden zusätzlich folgende Bereiche erfragt:

- frühere Diabetes-Schulung;
- soziodemographische Variablen: Schulbildung, Familienstand, Beruf.

Standardisierte Arztfragebögen:

• Rekrutierungsbogen:

Dieser Fragebogen wird vom behandelnden Arzt für alle Patienten bei Aufnahme in die Studie ausgefüllt. Insbesondere werden folgende Bereiche dokumentiert:

- ausführliche Krankengeschichte,
- Diabetes: Dauer der Erkrankung, bisherige Behandlung, Folgeerkrankungen,
- Komorbidität,
- Medikation bei Rekrutierung,
- Laboruntersuchung bei Rekrutierung,
- Art der Versorgung: Disease Management Programme oder „usual care“,
- Einschätzung der Compliance durch den Arzt.

• Verlaufsbogen:

Der behandelnde Arzt dokumentiert für alle Studienteilnehmer bei allen Folgeerhebungen u. a. folgende Bereiche:

- Neuerkrankungen (insbesondere Diabetes-Folgekrankheiten),
- Laboruntersuchungen (u.a. nüchtern Blutglucose, Lipide, Kreatinin, Albumin im Urin),

− Untersuchungsbefunde (u.a. Körpergewicht, Blutdruck, Pulsstatus, Sensibilität),

− Medikation,

− diabetesbedingte Überweisungen zur fachärztlichen Mitbehandlung (z.B. Augenärzte, Nephrologen),

− stationäre Krankenhausaufenthalte und deren medizinische Ursachen.

Zu Beginn der Studie werden von den teilnehmenden Praxen strukturelle Praxischarakteristika mittels eines standardisierten Fragebogens erhoben, insbesondere:

− Art der Praxis: Einzel- oder Gemeinschaftspraxis, Praxisgemeinschaft,

− Niederlassungsjahr,

− Zusatzqualifikationen,

− Scheinzahl, Anteil an Privatpatienten.

Studienteil B:

Die Patienten, die das zusätzliche Einschlusskriterium für Studienteil B erfüllen, werden entweder in einen der beiden Arme der Interventionsstudie randomisiert oder – falls randomisierungsunwillig – in die zweite Kontrollgruppe aufgenommen. In der Interventionsgruppe erfolgt das Telefoncounseling im Abstand von 4 - 6 Wochen während der ersten 12 Monate nach Rekrutierung. Die Interventions- und Kontrollgruppen werden zusätzlich nach 6, 12 und 18 Monaten per Fragebogen (standardisierter Patientenfragebogen - s.o.) befragt. Die durchgeführte Behandlung einschließlich der veranlassten diagnostischen und therapeutischen Maßnahmen sowie mögliche diabetesbedingte Folgeerkrankungen (z. B. Herzinfarkte) und Krankenhausaufenthalte werden nach Abschluss der Intervention nach 12 und 18 Monaten von den behandelnden Ärzten mittels des standardisierter Verlaufsbogen (s. o.) dokumentiert.

Nach Beendigung des Studienteils B werden auch diese Patienten im Rahmen der epidemiologischen Längsschnittuntersuchung langfristig weiter beobachtet.

Untersuchung von Blutparametern

Die Messung der primären Zielgröße (HbA_{1c}) und anderer Blutparameter erfolgt bei allen Studienteilnehmern (Studienteil A und B) bei Rekrutierung und den Folgeerhebungen

Bei den Teilnehmern des Studienteils B wird zusätzlich eine Messung der primären Zielgröße nach 12 Monaten (Ende Interventionsphase) durchgeführt.

Die Blutabnahme erfolgt in den Praxen. Insgesamt werden 30ml (EDTA + Serum) von den Patienten abgenommen. Das Handling der Blutproben in den Praxen, der Versand und die Analysen

des Zentrallabors im Raum Ludwigsburg sowie die Lagerung der Blutproben wird einheitlich gemäß „standard operating procedures“ erfolgen.

Die nicht unmittelbar für die HbA_{1c}-Bestimmung erforderlichen Teile der Blutproben werden zur wissenschaftlichen Erforschung weiterer Marker, die für die Entstehung und den Verlauf des Diabetes mellitus und möglicher Folgeerkrankungen von Bedeutung sein könnten (einschl. genetischer Marker), an die Abteilung Klinische Epidemiologie und Alternsforschung weitergeleitet und übereignet. Sie werden dort ohne zeitliche Begrenzung asserviert, um mit den verbesserten labortechnischen Möglichkeiten der Zukunft und unter Berücksichtigung zwischenzeitlicher neuer wissenschaftlicher Erkenntnisse zu späteren Zeitpunkten gezielte Laboranalysen durchführen zu können. Das biologische Material, das ohne zeitliche Beschränkung aufgehoben wird, wird nach Abschluss der Datenerhebung vollständig anonymisiert, so dass kein Rückschluss auf den individuellen Studienteilnehmer möglich ist. Gegebenenfalls werden für spezielle Laboranalysen, die nicht am DKFZ durchgeführt werden können, die Proben an externe Partner mit der entsprechenden Expertise auf dem jeweiligen Gebiet weitergeleitet, die im Rahmen einer wissenschaftlichen Kooperation in das Projekt eingebunden sind. Kommerzielle Nutzungsinteressen werden dabei ausgeschlossen.

8. Begleittherapie

Nicht relevant.

9. Sicherheitslabor (soweit einschlägig)

Nicht erforderlich.

10. Abbruchkriterien

10.1 Individuelle Abbruchkriterien

- Rücknahme des Einverständnisses
- Eintritt der Nicht-Teilnahmefähigkeit
- Bei Rücktritt von der Studie haben die betreffenden Teilnehmer das Recht, die Vernichtung der pseudonymisierten Angaben zu verlangen. Hierzu werden zum Zeitpunkt des Rücktritts diese Möglichkeiten des Widerrufs differenziert aufgelistet und erläutert sowie einzeln abgefragt.

10.2 Abbruchkriterien für die Gesamtstudie

- Mangelnde Kooperationsbereitschaft der Praxen

- Unzureichende Patientenrekrutierung
- Für den Interventionsteil der Studie ist keine Zwischenauswertung vorgesehen, die einen vorzeitigen Abbruch begründen könnte.

11. Statistisches Design

Studienteil A:

Die statistische Auswertung schließt insbesondere folgende Analysen ein:

- eine sorgfältige Beschreibung der Gesamtkohorte hinsichtlich diabetesrelevanter Parameter (u.a. Alter, Geschlecht, Dauer der Erkrankung, Medikation, Körpergewicht, Komorbidität, Lebensstilfaktoren) bei Rekrutierung,
- epidemiologische Analysen zu Determinanten und prognostischen Markern bei den Patienten in der Gesamtkohorte nach der Durchführung weiterer Follow-up Untersuchungen.

Studienteil B:

Powerberechnung:

Unter der Annahme, dass sowohl in der Interventions- als auch in der Kontrollgruppe im Verlauf der Erhebung jeweils 4 Patienten ausscheiden (n=120 in beiden Gruppen), bei zweiseitigem Testen, gleicher Varianz, einer Standardabweichung von 1,0, einem Signifikanzniveau von α=0,05, ergibt sich hinsichtlich der Senkung des HbA_{1C} folgende statistische Power:

**Senkung des HbA_{1C} um ... %-Punkte in der Interventionsgruppe**	**Power**
0,1	0,12
0,2	0,34
0,3	0,64
0,4	**0,87**
0,5	**0,97**
0,6	**0,99**
0,7	**1,00**
0,8	**1,00**
0,9	**1,00**
1,0	**1,00**

Die vorgeschlagene Studie besitzt folglich mit der gewählten Stichprobengröße ausreichende Po-

wer, um klinisch relevante Veränderungen des HbA_{1C} –Wertes statistisch signifikant nachweisen zu können.

Die wissenschaftliche Evaluation wird folgende Zielvariablen untersuchen:

Primäre Zielvariable: ***HbA_{1C}***

HbA_{1C} ist der wesentliche Zielparameter der Diabetesbehandlung und zeigt die Qualität der Stoffwechseleinstellung der letzten 3 Monate an.

HbA_{1C} wird zu Beginn der Studie und nach der Interventions- und Nachbeobachtungsphase bei allen Studienteilnehmern bestimmt.

Sekundäre Zielvariablen:

Der Diabetes mellitus ist eine chronische Erkrankung, bei der die weitere gesundheitliche Entwicklung (Auftreten von Komplikationen und Folgeerkrankungen) maßgeblich durch eine möglichst ganzheitliche medizinische Versorgung sowie Lebensstilfaktoren mitbestimmt wird. Aus diesem Grunde werden neben dem HbA_{1c} als primärer Zielvariable folgende weiteren sekundären Zielvariablen evaluiert:

- Fettstoffwechseleinstellung
- Blutdruck
- Körpergewicht, körperliche Aktivität, Rauchen
- gesundheitsbezogene Lebensqualität
- Inzidenz diabetes-assoziierter Folgeerkrankungen
- Krankenhausaufenthalte
- Sterblichkeit

Primäre Arbeitshypothese ist, dass sich durch ein supportives Telefoncounseling dieser Patienten der Zielparameter der Diabetestherapie (HbA_{1c}) verbessern lässt. Sekundäre Arbeitshypothese ist, dass sich als Folge auch sekundäre Outcome-Parameter (diabetesbedingte Folgeerkrankungen, Krankenhauseinweisungen, Sterblichkeit usw.) verbessern lassen.

Die statistische Auswertung schließt insbesondere folgende Analysen ein:

- eine sorgfältige Beschreibung und den Vergleich der Kontroll- und Interventionsgruppe hinsichtlich diabetesrelevanter Parameter (u.a. Alter, Geschlecht, Dauer der Erkrankung, Medikation, Körpergewicht, Komorbidität, Lebensstilfaktoren),
- eine sorgfältige Beschreibung und den Vergleich der Kontroll- und Interventionsgruppe hinsichtlich der primären und sekundären Zielvariablen am Ende der Interventionsphase,

- Analyse zur Assoziation zwischen der Intervention und der Entwicklung der Zielvariablen während der Nachbeobachtungsphase (Nachhaltigkeit der Intervention),
- eine sorgfältige Beschreibung und ein Vergleich der nicht randomisierungswilligen Patienten mit der Kontroll- und Interventionsgruppe hinsichtlich der primären und sekundären Zielvariablen am Ende der Interventionsphase (Einschätzung der externen Validität / Generalisierbarkeit der Resultate).

Als Hauptauswertung der Interventionsstudie (Studienteil B) wird der Vergleich der Änderung des HbA_{1c}-Wertes in beiden Studiengruppen mittels multipler linearer Regression unter Berücksichtigung potentieller Störgrößen durchgeführt.

Weitere Analysen werden ebenfalls unter sorgfältiger Berücksichtigung potentieller Störgrößen mittels multivariater Regressionsmodelle (je nach Outcome multiple lineare Regression bzw. Cox Proportional Hazards Modell) durchgeführt. Sie basieren auf der Grundlage von a-priori formulierten Analyseprotokollen. Alle Analysen werden mit dem Softwareprogramm SAS unter der Supervision des Projektleiters durchgeführt werden.

In Subgruppenanalysen wird auch besonderes Augenmerk auf genderspezifische Aspekte des geplanten Projekts gelegt, hierfür werden nach Geschlecht stratifizierte Auswertungen durchgeführt. So können in den zu bildenden multivariaten Modellen unterschiedliche Faktoren berücksichtigt werden, in denen sich Männer und Frauen unterscheiden (Gesundheitsverhalten, Wahrnehmung von Krankheit/Gesundheit, etc).

12. Ethische und rechtliche Aspekte

Die Untersuchung wird in Übereinstimmung mit der Deklaration von Helsinki in der aktuellen Fassung von 1996 sowie den auf dieser Deklaration aufbauenden, durch die Deutsche Arbeitsgemeinschaft für Epidemiologie (DAE) verabschiedeten Leitlinien für 'Good Epidemiologic Practice (GEP)' und gemäß der Berufsordnung für Ärztinnen und Ärzte der Landesärztekammer Baden-Württemberg in den jeweils aktuellen Fassungen durchgeführt. Die Teilnahme der Patienten an der Untersuchung ist freiwillig. Die Zustimmung kann jederzeit, ohne Angabe von Gründen und ohne Nachteile für die weitere medizinische Versorgung, zurückgezogen werden. Die Studie ist mit keinerlei Risiken für die Teilnehmer verbunden. Die Teilnehmer haben ausreichend Zeit, sich zu Hause mit dem Studieninhalt- und Ablauf auseinander zu setzen. Sie haben die Möglichkeit, Fragen an das Studiensekretariat oder die beteiligten Hausärzte zu richten. Nach Beantwortung eventueller Fragen durch das Studiensekretariat unterschreiben die zur Teilnahme bereiten Personen die Einverständniserklärung.

Die Patienten werden vor Studienbeginn schriftlich und mündlich über Wesen und Tragweite der

geplanten Untersuchung, insbesondere über den möglichen Nutzen für ihre Gesundheit und eventuelle Risiken, aufgeklärt. Ihre Zustimmung wird durch Unterschrift auf der Einwilligungserklärung dokumentiert. Bei Rücktritt von der Studie hat der Patient das Recht, die Vernichtung bereits gewonnenen (Daten-) Materials zu verlangen.

Der Studienplan wird vor Studienbeginn der Ethikkommission der Medizinischen Fakultät Heidelberg zur Begutachtung vorgelegt. Es wird nicht mit dem Einschluss von Patienten / Probanden begonnen, bevor nicht das schriftliche, zustimmende Votum der Ethikkommission vorliegt.

Die Namen der Patienten und alle anderen vertraulichen Informationen unterliegen der ärztlichen Schweigepflicht und den Bestimmungen des Bundesdatenschutzgesetzes (BDSG). Eine Weitergabe von Patientendaten erfolgt ggf. nur in pseudonymisierter Form. Dritte erhalten keinen Einblick in Originalkrankenunterlagen. Kommerzielle Nutzungsinteressen werden dabei ausgeschlossen.

Patientenfragebögen, Einverständniserklärung und Übereignungsvertrag

Die von den Studienteilnehmern auszufüllenden Fragebögen sind mit einer eindeutigen Identifikationsnummer versehen. Die Studienteilnehmer werden gebeten, auf dem Deckblatt des Fragebogens Namen, Adresse und Geburtsdatum einzutragen. Nach Eingang des Fragebogens am DKFZ wird das Deckblatt mit den personenbezogenen Daten vom restlichen Fragebogen, der ausschließlich die Identifikationsnummer enthält, abgetrennt. Die weitere Aufbewahrung und Verarbeitung der personenbezogenen Daten erfolgt vollkommen getrennt von der Verarbeitung der Studiendaten.

Die auf dem Deckblatt vorhandenen personenbezogenen Daten werden auf einem im Studiensekretariat stehenden, nicht vernetzten Rechner (Stand-Alone-PC) verarbeitet. Auf diesem Rechner befinden sich die Programme sowie die Dateien zur Verwaltung der StudienteilnehmerInnen. Zugriff auf diesen Rechner haben ausschließlich die mit der Studienorganisation beauftragten Personen. Der Rechner ist durch eine spezielle Sicherheitssoftware (Safeguard Easy) gesichert, für die ein spezielles Kennwort benötigt wird, um Zugang zu erhalten.

Das Deckblatt des Fragebogens wird nach Entfernen der Identifikationsnummer ausschließlich in einem gesonderten Stahlschrank in einem Raum mit Sonderschließanlage im Studiensekretariat aufbewahrt. Nur ein begrenzter und namentlich zu benennender Personenkreis besitzt einen Schlüssel zu diesem Raum.

Die Einverständniserklärung und der Übereignungsvertrag enthalten Name und Unterschrift des Studienteilnehmers. Sie werden nach Eingang am DKFZ genauso wie für das Deckblatt des Fragebogens beschrieben (s.o.) aufbewahrt

Blutproben

Die Blutproben werden ausschließlich in pseudonymisierter Form (nach Abschluss der Datenerhebung in anonymisierter Form), nur mit der Identifikationsnummer versehen, gesammelt, transportiert, gelagert und bearbeitet / analysiert.

Unterschriften

Prof Dr. med. H. Brenner Dr. med. Elke Raum

Literatur

(1) Rothenbacher D, Brenner H, Rüter G. Netreuung von chronisch Kranken in der Hausarztpraxis am Beispiel des Typ 2 Diabetes. Dtsch Ärztebl 2005;102:A 2408-2412.

(2) Nationale Versorgungs-Leitlinie Diabetes mellitus Typ-2. URL: http://www.versorgungsleitlinien.de/themen/diabetes2 (letzter Zugriff: 16.04.2008).

(3) Rathmann W, Haastert B, Icks A, Herder C, Kolb H, Holle R, et. Al. Die Diabetes-Epidemie in der älteren Bevölkerung in Westeuropa: Daten aus bevölkerungsbezogenen Studien. Gesundheitswesen 2005;67 (S1):S110-S114.

(4) Stock S, Redaelli M, Wendland G, Civello D, Lauterbach KW. Diabetes-prevalence and cost of illness in Germany: a study evaluating data from the satutory health insurance in Germany. Diabet Med 2006;23:299-305.

(5) Icks A, Rathmann W, Haastert B, Mielck A, Holle R, Löwel H, et. al. Versorgungsqualität und Ausmaß von Komplikationen an einer bevölkerungsbezogenen Stichprobe von Typ 2-Diabetespatienten. Dtsch Wochenschr 2006;131:73-78.

(6) International Diabetes Foundation. International standards for diabetes education 2003. http://www.idf.org/webdata/docs/International%20standards.pdf (letzter Zugriff: 16.04.2008).

(7) Campbell SM, Hann M, Hacker J, Burns C, Oliver D, Thapar A, et. al. Identifying predictors of high quality care in English general practice: observational study. BMJ 2001;323:1-6.

Appendix

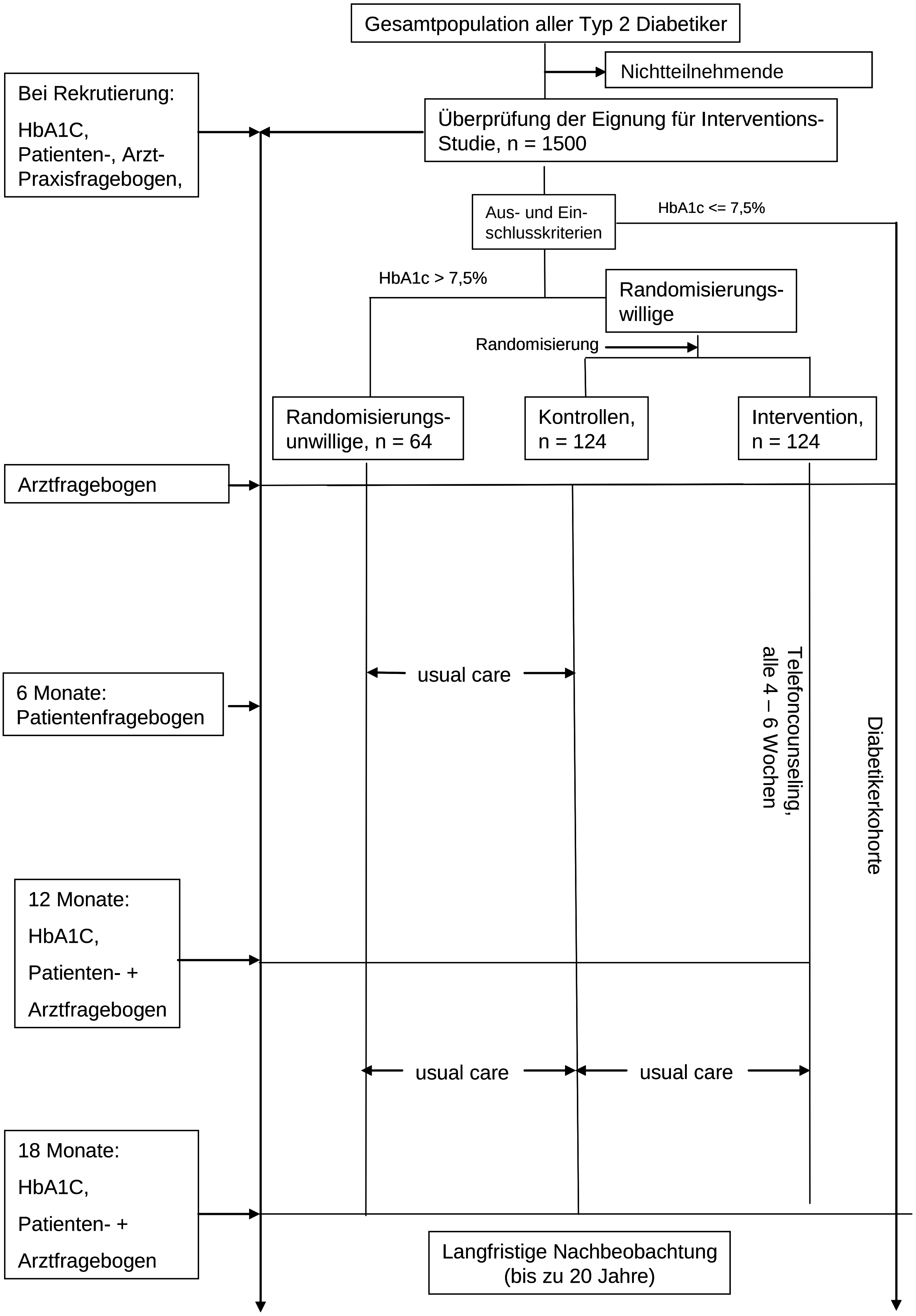
Gesamtpopulation aller Typ 2 Diabetiker
Nichtteilnehmende
Bei Rekrutierung:
HbA1C,
Patienten-, Arzt-
Praxisfragebogen,
Überprüfung der Eignung für Interventions-Studie, n = 1500
Aus- und Ein-schlusskriterien
HbA1c <= 7,5%
HbA1c > 7,5%
Randomisierungs-willige
Randomisierung
Randomisierungs-unwillige, n = 64
Kontrollen, n = 124
Intervention, n = 124
Arztfragebogen
usual care
Telefoncounseling, alle 4 – 6 Wochen
Diabetikerkohorte
6 Monate:
Patientenfragebogen
12 Monate:
HbA1C,
Patienten- +
Arztfragebogen
usual care
usual care
18 Monate:
HbA1C,
Patienten- +
Arztfragebogen
Langfristige Nachbeobachtung (bis zu 20 Jahre)
